# Surgical management of achalasia

**DOI:** 10.1007/s00261-024-04664-3

**Published:** 2024-11-25

**Authors:** Margaux Mustian, Kristen Wong

**Affiliations:** 1 University of Alabama at Birmingham, Birmingham, USA; 2 Birmingham VA Medical Center, Birmingham, USA

**Keywords:** Achalasia, Surgery, Heller myotomy, POEM

## Abstract

Achalasia is a chronic esophageal motility disorder comprised of ineffective esophageal peristalsis and incomplete relaxation of the lower esophageal sphincter. This disease had historically been managed through medical means as well as endoscopic dilations. However, surgical interventions are now considered standard of care, including minimally invasive Heller myotomy, which was popularized in 1990s, followed by per oral endoscopic myotomy in the 2010s. Both surgical approaches provide acceptable resolution of dysphagia symptoms. Classification of the achalasia as well as other patient-level factors may drive the clinical decision-making between the two approaches, as well as surgical training and surgeon preference.

## Introduction

Achalasia is comprised of failure of lower esophageal sphincter (LES) relaxation and lack of coordinated esophageal peristalsis [1]. Symptoms typically include dysphagia, cough, chest pain, vomiting, and weight loss, which can be severely debilitating [2]. Although the disease process is not typically life-threatening, it can substantially impact patients’ quality of life. Left untreated achalasia can lead to decompensated sigmoid esophagus and malnutrition [2]. Additionally, prolonged history of achalasia is associated with greater than 50-fold risk of esophageal adenocarcinoma and also increased risk for esophageal squamous cell carcinoma [2].

There are several treatment options available for patients living with this chronic disease, including several surgical interventions. The two most commonly offered surgical treatment options for achalasia in the United States include minimally invasive Heller myotomy and per oral endoscopic myotomy (POEM). The two surgical interventions differ in approach, as minimally invasive Heller myotomy is a transabdominal approach through laparoscopic incisions, while POEM is an incision-less procedure performed through an endoscope. However, both of these interventions target the LES with the goal of providing symptom improvement. This is accomplished by completely dividing the muscular fibers of the LES, thus allowing a bolus to pass through the esophagogastric junction.

## Preoperative work up

Prior to surgical intervention for achalasia, a thorough work up is necessary [including endoscopy, barium esophagram, and high resolution manometry (HRM)] to confirm the diagnosis and rule out other etiologies for dysphagia, including malignancies, strictures or other esophageal motility disorders [3]. HRM aids in further classifying achalasia (Chicago Classification v4.0) and can help determine surgical approach and predict efficacy of the surgical intervention. Chicago Classification v4.0 classifies achalasia as either type I, type II, or type III achalasia on the basis of HRM findings) [4]. Type I is comprised of increased intergrade relaxation pressure (IRP) and absence of esophageal contractility [4]. Type II is characterized by increased IRP and pan-esophageal pressurization with pan-esophageal pressure in at least 20% of swallows [4]. Type III consists of premature and/or spastic contractions, with an elevated IRP and at least 20% premature contractions [4]. Based on this classification, certain types of achalasia have varying treatment responses to one modality or another, which will be described in greater detail later in this article. Importantly, both POEM and minimally invasive Heller myotomy offer palliation of symptoms but do not completely eradicate the disease process.

Determining preoperative symptomatology is also important, and can be assessed through a variety of validated surveys. The most commonly used is the Eckardt scoring system, which focuses on frequency of chest pain, regurgitation, dysphagia and weight loss [5]. Other surveys assess the impact of achalasia symptoms on the patients’ quality of life and include: Mayo Dysphagia Questionnaire, Achalasia Disease-specific Quality of Life Measure, and Visceral Sensitivity Index. Documentation of symptoms can be especially helpful in assessing efficacy of the surgical intervention.

## Heller myotomy

### Adoption of Heller myotomy

Historically, pneumatic dilation had been the mainstay of treatment for achalasia patients prior to the adoption of minimally invasive surgery [6]. Although the open Heller myotomy was originally performed in 1914 [7], this approach was typically reserved for failure of dilation or esophageal perforation during dilatation [8]. However, in the early 1990s, a minimally invasive transthoracic esophagomyotomy was first described [9]. This evolved into a transabdominal laparoscopic approach in 1992, and the laparoscopic Heller myotomy with partial fundoplication became the gold standard surgical approach for treatment of achalasia shortly thereafter [10]. The Heller myotomy operation has also been adopted using robotic approaches [11] as well as via laparoscopic single-site access [12].

### Operative approach: laparoscopic Heller myotomy

**Fig. 1:**
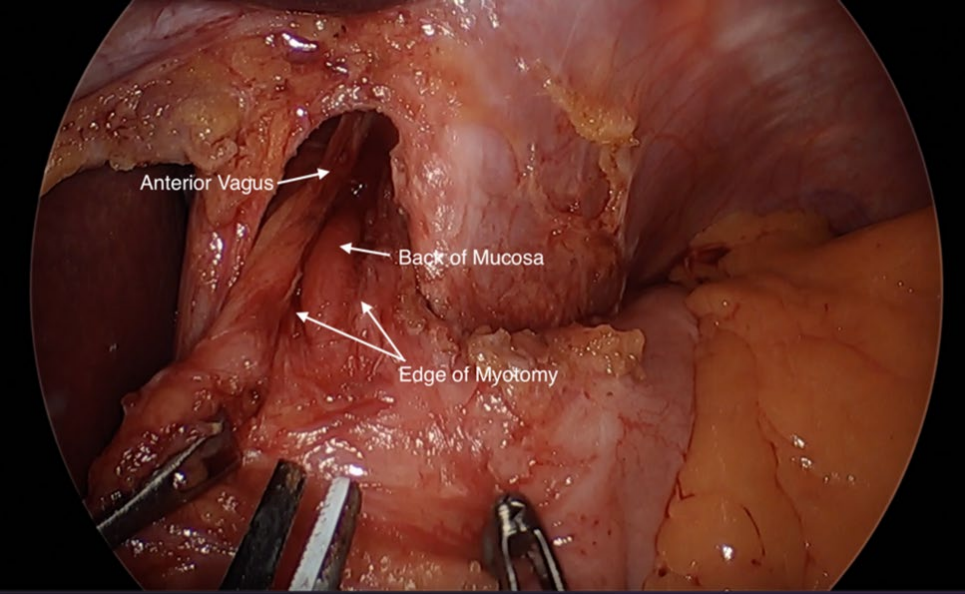
Preservation of the anterior vagus nerve during mediastinal dissection for minimally invasive Heller myotomy [42]

**Fig. 2:**
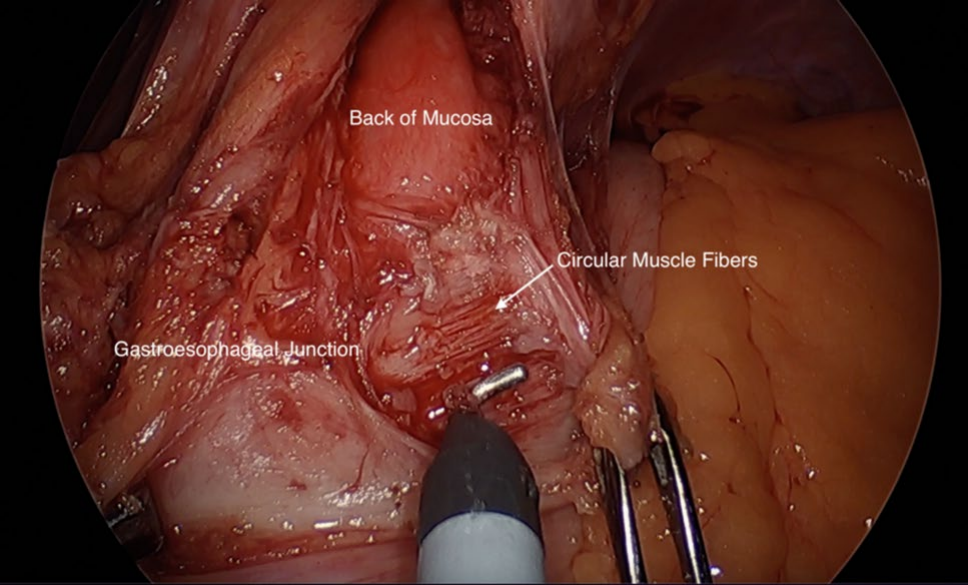
Minimally invasive Heller myotomy with disruption and division of the circular muscular fibers [42]

The operation begins with access to the abdomen and retraction of the liver in order to visualize the stomach and hiatus. The short gastric vessels are then divided and the right and left crus are identified. Mediastinal dissection is completed anteriorly until adequate esophageal length is achieved, taking care to preserve the anterior vagus nerve (Fig. 1). The anterior myotomy path is then mapped with a goal of achieving a myotomy of at least 4–5 cm (cm) in length on the esophagus and extending down 2–3 cm onto the stomach [13]. The longitudinal fibers are split and the circular muscle are divided until the back of the mucosa is visualized (Fig. 2). During this division of muscle fibers, the gastroesophageal junction is the most likely place for inadvertent mucosal injury [13].

**Fig. 3:**
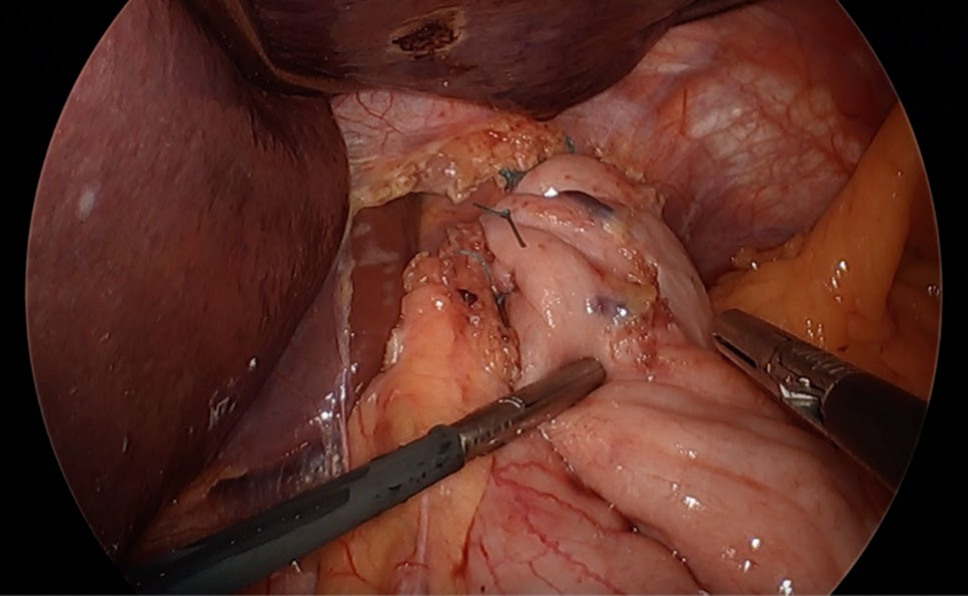
Anterior (Dor) fundoplication following myotomy [42]

The biggest concern after myotomy is the development of gastroesophageal reflux. Therefore, a fundoplication is usually performed at the same time. As the goal of the operation is to permit passage of bolus through the LES, complete wraps (Nissen 360 degree fundoplications) are typically avoided, as they are also associated with the risk of developing dysphagia. Instead, partial wraps, including both Toupet (270° posterior wrap) and Dor (180° anterior) fundoplications (Fig. 3) are routinely utilized in combination with Heller myotomy, depending on surgeon preference. There is some debate regarding fundoplication selection between these two partial wraps. Advocates for the Dor fundoplication cite that it requires less mobilization and disruption of posterior esophageal attachments [14]. Additionally, if during the myotomy, an esophageal mucosal injury occurs, it should be repaired at the time of the operation and also then buttressed with an anterior Dor fundoplication, adding another anterior layer of protection over the mucosal defect [14]. On the other hand, in the absence of a musosal injury, there is a theoretical added benefit of “stenting” open the myotomy with the posterior 270° wrap (Toupet), which then may lead to less recurrent dysphagia [13, 14]. Regardless, there is no clear evidence for one partial fundoplication over another (Dor vs. Toupet) in the literature, as the data are lacking and have mixed results [15], 16].

### Contraindications and complications

Contraindications for minimally invasive Heller myotomy with partial fundoplication include medical comorbidities that prohibit use of pneumoperitoneum during laparoscopic or robotic surgery.

Potential immediate complications after Heller myotomy include pneumothorax, bleeding, esophageal and stomach perforations and leaks. Typically, esophageal perforations can be primarily repaired if discovered intraoperatively and covered with anterior partial fundoplication (Dor). If not discovered at the time of operation, they can lead to leak with or without intra-abdominal sepsis, and require intervention such as endoscopic esophageal stent placement (if patient is stable) or laparoscopic vs. open abdominal washout and drain placement. Long term complications include gastroesophageal reflux disease leading to erosive esophagitis. Gastroesophageal reflux disease (GERD) develops in approximately 20–30% of people after Heller myotomy, though this risk decreases with fundoplication being performed [17]. The risk of reflux following Heller myotomy is due to the disruption of the body’s natural anti-reflux barriers including the LES, the sling fibers of the stomach, and the phrenoesophageal ligament.

Recurrent symptoms of achalasia or dysphagia may also occur postoperatively. This may be due to incomplete myotomy or due to the chronic nature of the disease process. Repeat laparoscopic Heller myotomy may be considered if recurrent symptoms appeared to be due to technical failure, but alternative therapeutic strategies including POEM may better serve patients with recurrent disease [18]. Pneumatic dilations can also be used to treat recurrent dysphagia symptoms with excellent success rates [19].

## POEM

### Adoption of POEM

The first endoscopic myotomy for achalasia in a human was performed in 2008 in Japan [20]. Since then, it has rapidly gained popularity in the United States due to its proven safety and efficacy [21]. Based on a randomized clinical trial non-inferiority study, the procedure has been shown to be non-inferior to laparoscopic Heller myotomy in regards to symptom relief for type I and II achalasia subtypes[21], and it has improved clinical outcomes for type III achalasia because of the ability to perform a longer esophageal myotomy [22]. Despite its popularity, POEM tends to be only offered at large academic centers by gastroenterologists or surgeons with fellowship training in third space endoscopy.

### Operative approach: POEM

**Fig. 4:**
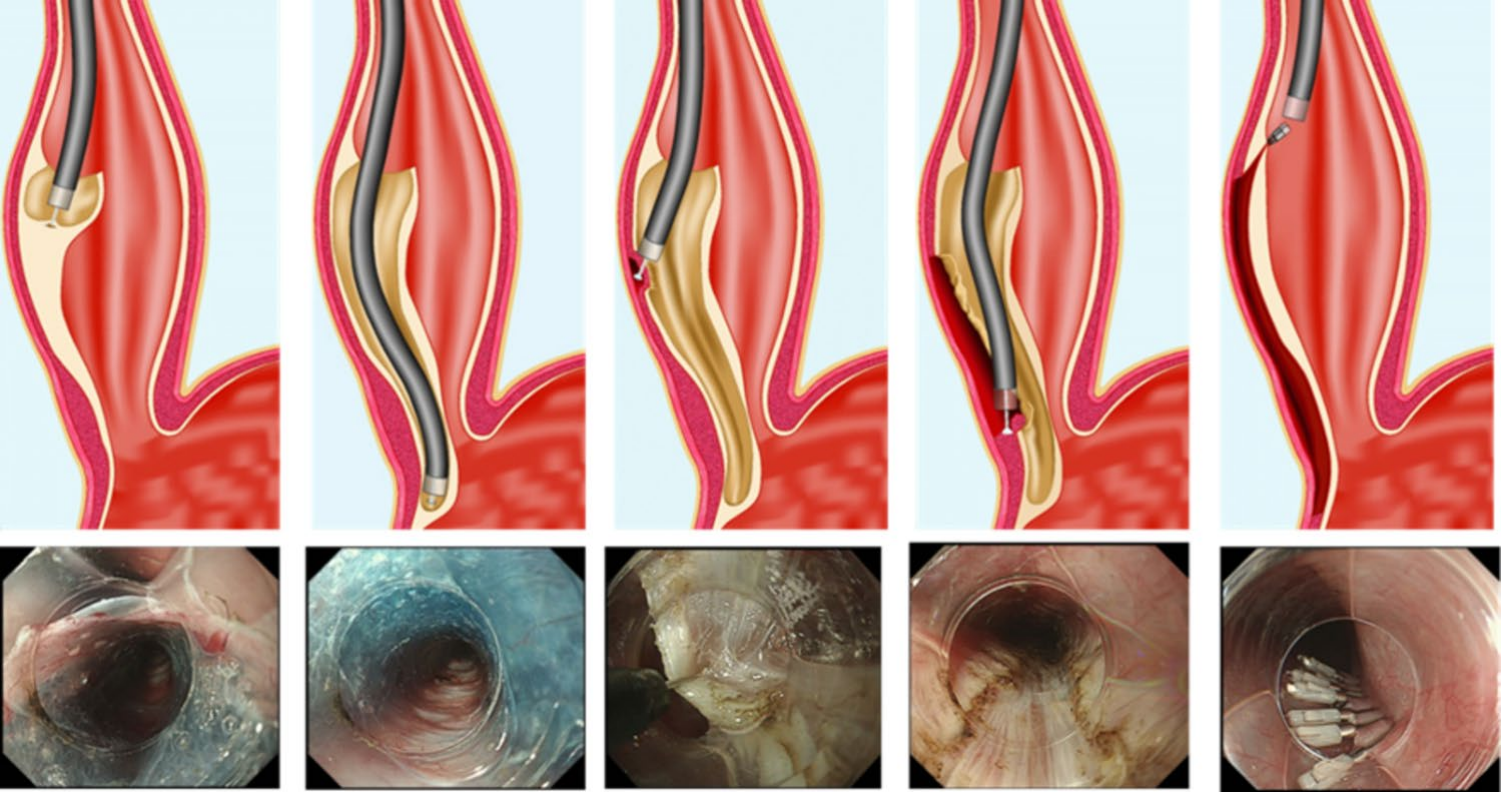
Per oral endoscopic myotomy (POEM): creation of a submucosal lift and submucosal tunnel followed by myotomy and clip closure of the mucosotomy [43] Digestive Diseases Center, Showa University Koto Toyosu Hospital

The procedure begins with a diagnostic esophagogastroduodenoscopy to rule out ulcers, esophagitis, or candida infection. A clear, distal end cap attachment is placed onto the end of the scope to give an unobstructed view once ready to create the tunnel. A site is then chosen for the mucosotomy which can be done on the anterior or posterior side of the esophagus, 6–12 cm proximal to the gastroesophageal junction (length of myotomy can be tailored to preoperative manometry findings). A submucosal lift with a mixture of methylene blue or indigo carmine diluted with normal saline is performed using an endoscopic injection needle. A longitudinal 2 cm mucosotomy is created using an endoscopic cautery knife (Fig. 4). The submucosal tunnel is entered and extended to approximately 3 cm beyond the high pressure zone of the gastroesophageal junction. The circular muscle myotomy is performed starting distal to the mucosotomy and carried in an antegrade fashion through the LES and at least 2–3 cm onto the gastric side [23]. The extent of myotomy can be confirmed with the double endoscope technique using transillumination within the tunnel and a second retroflexed pediatric endoscope in the stomach [24], or a submucosal lift that can be seen from the true lumen on the gastric side. Once complete, the endoscope is withdrawn from the tunnel and the mucosotomy is closed using through the scope hemoclips or endoluminally sutured.

### Contraindications and complications

Contraindications to a per oral endoscopic myotomy include severe erosive esophagitis, previous endoscopic mucosal resection or ablation, previous radiation to the esophagus, portal hypertension, or significant coagulation disorders [25].

Perioperative complications for the POEM procedure are rare. Serious adverse events that have been described are mucosal injury leading to leak and bleeding (< 2%) [23]. If they occur, both would likely require reintervention. Minor adverse effects include capnothorax or pneumoperitoneum (which resolve on their own), subcutaneous emphysema, chest or abdominal pain, fever.

The most important long-term complication of POEM is GERD. There are high rates of post-POEM GERD reported in the literature, ranging from clinical symptoms of GERD in 30–40%, objective, abnormal pH studies in around 50–60%, and endoscopic evidence of severe GERD such as Grade C or D esophagitis in 15–20% [24, 26]. Fortunately, most of the patients who develop GERD post POEM have symptoms well controlled on a proton pump inhibitor [27]. There is concern that younger patients may be at higher risk for Barrett’s esophagus and dysplasia over time, due to years of pathologic acid exposure in the esophagus. It is recommended to perform surveillance endoscopy every 2–3 years on anyone undergoing POEM.

## Postoperative management: Heller myotomy and POEM

Following Heller myotomy or POEM, patients are generally admitted to the floor after discharge from the post anesthesia care unit. Some surgeons or endoscopists keep the patient nil per os (NPO) for the night of surgery while others initiate a clear liquid diet on postoperative day zero [28]. Some centers and/or surgeons will routinely obtain postoperative imaging for patients on postoperative day 1 to assess for leak and/or passage of oral contrast, while others only image for concerning features (intraoperative concerns, postoperative tachycardia or other clinic findings) [13, 28]. Clear liquid diet is then initiated and then advanced to full liquid diet for 2 weeks followed by a soft diet [13]. Patients should be seen in follow up several weeks postoperatively to assess improvement in symptoms following the intervention. If patients do not have symptoms of dysphagia., it is recommended to avoid re-imaging for 6 months if possible, as postoperative changes may account for subtleties in findings [28].

## Comparison of outcomes: Heller myotomy vs. POEM

A systematic review from 2018, including 74 studies, found that mean follow up was longer for patients following Heller myotomy than POEM, but POEM was associated with greater predicted probability for improvement in dysphagia symptoms at both 12 and 24 months [29]. However, both Heller myotomy and POEM had greater than 90% probability of dysphagia improvements at both time points [29]. POEM patients were also found to have higher likelihood of developing GERD (OR 1.69; 95% CI 1.33–2.14; p < 0.0001) [29].

A randomized clinical trial also demonstrated that POEM and Heller myotomy patients had similar symptom improvement at short and longer term (12 month) follow up; however, POEM patients had both shorter anesthesia and procedure times [30]. Another study comparing outcomes of Heller myotomy and POEM patients at a single institution found that POEM patients had shorter duration of narcotic use postoperatively and quicker return to normal activities of daily life [27].

## Selection of treatment: patient considerations

The initial surgical treatment selection for achalasia may be largely based on patients’ HRM findings and Chicago classification. Society of American Gastrointestinal and Endoscopic Surgeons (SAGES) guidelines recommend either POEM or Heller myotomy for treatment of type I and II achalasia, depending on surgeon experience and/or preference, in shared decision-making with the patients. However, for type III achalasia, the guidelines recommend that POEM be considered instead of Heller myotomy [31]. In a multicenter trial for patients with type III achalasia, it was demonstrated that the median myotomy length following POEM was 16 cm, compared with median 8 cm for Heller myotomy, which may serve type III achalasia better due to the much higher proximal extent of the myotomy made possible via endoscopic approach [22]. The authors also found that there was better clinical response to POEM than Heller for patients with type III achalasia, with Eckhardt scores ≤ 1 for 98.0% of POEM patients vs 80.8% of Heller patients [22].

Other patient considerations in selection of treatment for achalasia include prior treatment history. Those with scarring of the LES or prior endoscopic interventions including endoscopic mucosal resections may make POEM more challenging, which may make Heller myotomy a better approach. Likewise, those patients with concomitant hiatal hernia may benefit from minimally invasive myotomy where the hiatal hernia can be addressed in the same operation. During the endoscopic approach, the myotomy may be performed either anteriorly or posteriorly, whereas the Heller myotomy is performed on the anterior side of the esophagus. Therefore, for patients who have had recurrence of dysphagia symptoms following Heller myotomy, a posterior POEM is the treatment of choice. Additionally, as reflux symptoms may be worse following POEM compared with Heller myotomy, patients with preoperative reflux symptoms should be counseled regarding this increased risk of worsening GERD symptoms with POEM when selecting an operative intervention.

For patients that are poor surgical candidates due to medical comorbidities, endoscopic myotomy may not offer an advantage as patients still have to tolerate general anesthesia for POEM. However, POEM may be offered to patients with only contraindication to abdominal insufflation or those with hostile abdomens from multiple prior abdominal operations.

## Other interventions for achalasia

Aside from surgical operations and medical therapy, additional interventions are available for patients with achalasia including pneumatic balloon dilations and botulinum toxin (BT) injections. A large multicenter randomized clinical trial compared treatment naïve achalasia patients who were randomized to either POEM or pneumatic dilation, and it was found that patients undergoing POEM had higher reported treatment success at 2 years (92% vs. 54%, based on Eckhardt scores ≤ 3) with fewer adverse outcomes [32]. However, pneumatic dilations remain the most effective non-surgical treatment option for achalasia [33]. Due to the 1.9% risk of perforation associated with pneumatic dilations, these procedures should only be performed among patients who are surgical candidates [33].

Along with oral medical therapy, BT injections are also used at the LES to aid in relief from dysphagia symptoms for patients with achalasia. Although BT injections have been shown to provide greater symptomatic relief compared with placebo, the therapeutic benefit is short term (typically several months), and patients usually require multiple treatments [34, 35]. BT is therefore usually reserved for elderly patients or those who are not good candidates for other operative interventions, such as surgery or pneumatic dilations. However, there are reports of surgical management of achalasia following BT injections, with some evidence of worse postoperative outcomes following myotomy in setting of prior BT injections [36].

## Surgical treatment for other esophageal motility disorders

In addition to the treatment of achalasia, POEM and minimally invasive Helller myotomy have also been performed for other esophageal motility disorders. Again, the use of imaging and endoscopy to rule out malignancy remains very important. The utilization of POEM for esophageal motility disorders aside from achalasia has been described for diffuse esophageal spasm and hypercontractile esophagus [37]. For esophageal motility disorders where intervention is not only needed at the the LES, POEM may provide better relief than Heller due to the proximal extent of myotomy. For these disorders it may be challenging to reach proximally enough from transabdominal (Heller) approach. However, the data are not robust regarding outcomes for surgical management of other primary esophageal motility disorders, compared with other nonsurgical approaches [38–40]. For esophagogastric junction outflow obstruction, surgical intervention could be considered depending on the underlying etiology of the obstruction, but the data currently remain limited for this as well [41].

## Conclusions

In summary, both POEM and minimally invasive Heller myotomy remain appropriate surgical interventions for achalasia, as well as some other primary esophageal motility disorders. Some patient-level factors or patient history may necessitate the selection of one approach over another. However, for the treatment of type I or II achalasia, in the absence of a clear contraindication for one operation or another, the operative treatment selection remains at the discretion of the patient and surgeon, with nearly equivocal postoperative outcomes. However, for patients with type III achalasia, POEM may provide better symptom relief.

## Data Availability

No datasets were generated or analysed during the current study.
